# Comparison of serological and molecular panels for diagnosis of vector-borne diseases in dogs

**DOI:** 10.1186/1756-3305-7-127

**Published:** 2014-03-26

**Authors:** Ricardo G Maggi, Adam J Birkenheuer, Barbara C Hegarty, Julie M Bradley, Michael G Levy, Edward B Breitschwerdt

**Affiliations:** 1 Vector Borne Disease Diagnostic Laboratory and the Intracellular Pathogens Research Laboratory, Center for Comparative Medicine and Translational Research, College of Veterinary Medicine, North Carolina State University, 1060 William Moore Dr., Raleigh, North Carolina 27607, USA

**Keywords:** Canine vector-borne diseases, Serology, Molecular testing, Diagnostic panel

## Abstract

**Background:**

Canine vector-borne diseases (CVBD) are caused by a diverse array of pathogens with varying biological behaviors that result in a wide spectrum of clinical presentations and laboratory abnormalities. For many reasons, the diagnosis of canine vector-borne infectious diseases can be challenging for clinicians. The aim of the present study was to compare CVBD serological and molecular testing as the two most common methodologies used for screening healthy dogs or diagnosing sick dogs in which a vector-borne disease is suspected.

**Methods:**

We used serological (*Anaplasma* species, *Babesia canis*, *Bartonella henselae*, *Bartonella vinsonii* subspecies *berkhoffii*, *Borrelia burgdorferi*, *Ehrlichia canis*, and SFG *Rickettsia*) and molecular assays to assess for exposure to, or infection with, 10 genera of organisms that cause CVBDs (*Anaplasma*, *Babesia*, *Bartonella*, *Borrelia*, *Ehrlichia*, *Francisella*, hemotropic *Mycoplasma*, *Neorickettsia*, *Rickettsia*, and *Dirofilaria*). Paired serum and EDTA blood samples from 30 clinically healthy dogs (Group I) and from 69 sick dogs suspected of having one or more canine vector-borne diseases (Groups II-IV), were tested in parallel to establish exposure to or infection with the specific CVBDs targeted in this study.

**Results:**

Among all dogs tested (Groups I-IV), the molecular prevalences for individual CVBD pathogens ranged between 23.3 and 39.1%. Similarly, pathogen-specific seroprevalences ranged from 43.3% to 59.4% among healthy and sick dogs (Groups I-IV). Among these representative sample groupings, a panel combining serological and molecular assays run in parallel resulted in a 4-58% increase in the recognition of exposure to or infection with CVBD.

**Conclusions:**

We conclude that serological and PCR assays should be used in parallel to maximize CVBD diagnosis.

## Background

When dogs develop clinical and hematological abnormalities, such as fever, cytopenias, hypoalbuminemia, hyperglobulinemia, polyarthritis or protein-losing nephropathy, veterinarians will often include vector-borne diseases among the differential diagnoses. In North America the number of recognized CVBDs has increased and now includes anaplasmosis (*Anaplasma phagocytophilum* or *Anaplasma platys*), babesiosis (*Babesia canis* and *Babesia gibsoni*), bartonellosis (numerous *Bartonella* sp.), heartworm disease (*Dirofilaria immitis*), ehrlichiosis (*Ehrlichia canis*, *Ehrlichia chaffeensis*, *Ehrlichia ewingii*, or *Ehrlichia muris*), hepatozoonosis (*Hepatozoon canis* and *Hepatozoon americanum*), Lyme disease (*Borrelia burgdorferi*), tularemia (*Francisella tularensis*), hemotropic *Mycoplasma* (multiple *Mycoplasma* sp.), Potomac Horse Fever (*Neorickettsia risticii*), and Rocky Mountain spotted fever (*Rickettsia rickettsii*). In addition, CVBDs such as *E. ewingii* and *E. chaffeensis* appear to have increased in incidence and geographic distribution due to the spread of *Ambylomma americanum* ticks northward throughout much of the United States. Also, *B. gibsoni*, which is frequently found in American Pitbull Terriers, can be transmitted directly to other dogs via fighting and bites from an infected animal [1] thus, this historically vector-borne pathogen can be directly transmitted among dogs. Although the mode(s) of transmission are incompletely understood, several *Bartonella* species are thought to be transmitted to dogs by fleas and ticks. These and other factors have contributed to an evolving appreciation of the role of CVBDs as a cause of disease in dogs. As the optimal treatment modalities differ for these diseases, it behooves veterinarians to choose CVBD diagnostic tests wisely, so as to economically and accurately evaluate exposure to and/or infection with a spectrum of vector-borne pathogens.

All diagnostic tests have inherent advantages and limitations. Serology relies on an immunologically appropriate and diagnostically detectable host immune response against one or more CVBD pathogens. As antibodies can persist for variable intervals after a pathogen is immunologically or therapeutically eliminated, serology does not confirm active or persistent infection in the patient, which is a diagnostic disadvantage. However, serology can be used to retrospectively confirm recent infection, by demonstrating seroconversion (i.e. a four-fold change in the patient’s antibody titer between acute and convalescent serum samples) [1]. The persistence of circulating antibodies can also be an advantage of serology in that antibodies may be detectable during chronic intravascular CVBD infections, when a pathogen may be circulating below the limit of PCR detection or sequestered in tissues that are not routinely submitted for PCR testing. Another potential limitation of serology includes diminished specificity, due to antibody cross-reactivity within or between CVBD genera [2-8]. Nevertheless, cross-reactivity between *Anaplasma*, *Bartonella*, *Ehrlichia*, and *Rickettsia *genera in dogs seems to be very unlikely, as dogs experimentally-infected with these pathogens develop very specific antibodies that do not cross react among genera [9-15].

Conversely, serological antigens chosen or available for assays may be too specific or mismatched to the etiological pathogens resulting in false negative results. These factors can result in the inability to accurately identify the infecting species or strain, which can have therapeutic implications for the patient. A technical limitation for the development of some CVBD serological assays is the inability to produce antigens in sufficient quantities to be used in indirect fluorescent antibody (IFA) or enzyme-linked immunoabsorbent assays (ELISA) (examples include *A. platys*, *E. ewingii*, *Babesia* and *Hepatozoon* spp.), although the use of synthetic peptides may help overcome this limitation. With the advent of PCR testing, it has also become obvious that some dogs do not mount a detectable antibody response, despite persistent intravascular infection with CVBD pathogens [16-20].

Similar to serology, PCR has advantages and disadvantages for the diagnosis of CVBDs. PCR has the distinct advantage over serology of detecting “active” infection in a single sample from a single time-point. Additionally, PCR can be used to specifically target a pathogen at the species or strain level by using different PCR primer sets or by sequencing PCR products. While some *a priori* knowledge or assumptions about the DNA sequence of a pathogen are needed to design PCR-based assays, PCR does not require definitive knowledge of the pathogen DNA sequence. Additionally, PCR does not require that the pathogen(s) be isolated or their antigens produced to achieve the development and validation of an assay. Multiple pathogens or species can be detected using multiplex PCR assays [21-23] but these assays can be more challenging in the context of achieving optimal sensitivity for all pathogens targeted in the panel. Co-infecting pathogens may cause competition in the PCR reaction process [24,25]. Substantially higher concentrations of one pathogen compared to the other(s) can result in detection of only one organism despite the presence of a co-infection. The main limitation to PCR testing is the requirement for adequate template (nucleic acid of the target organism) in the patient sample to achieve amplification of the target DNA sequence. For vector-borne pathogens such as *Anaplasma spp*., *B. burgdorferi*, and *Bartonella* spp., it is well documented that the number of intravascular organisms fluctuates over time following transmission [10,11,13,14]; therefore, PCR testing at a single time point may produce a false negative result for an infected patient. Some other technical disadvantages of PCR-based testing include potential false negative results due to the presence of PCR inhibitors that were not removed during the nucleic acid purification process and the potential for laboratory contamination resulting in false positive reactions in patients that are not infected. The latter disadvantages can be minimized by the use of appropriate techniques, reagents and the incorporation of appropriate controls. Unless mechanisms are developed to overcome all of the limitations of PCR-based testing, PCR is unlikely to serve as a stand-alone assay for the diagnosis of many vector-borne infections.

In the current study, a panel of serological assays was compared to multiple PCR assays using samples from healthy dogs and dogs suspected of being infected with one or more CVBDs.

## Methods

### Sample group selection

Paired serum and ethylenediaminetetraacetic acid (EDTA)-anti-coagulated blood samples from several defined groups of sick dogs were selected from a convenience sample of diagnostic accessions of the Vector-Borne Disease Diagnostic Laboratory (VBDDL), at the College of Veterinary Medicine, North Carolina State University. To facilitate a comparative analysis between serology and PCR, all patient samples were tested in parallel and in an identical manner, regardless of the test(s) originally requested by the attending clinician. In addition, paired serum and EDTA samples retrieved from storage at -80°C from 30 clinically healthy dogs examined during routine wellness appointments (Group I) were tested to establish background exposure to or infection with the defined population of CVBDs targeted in this study. Samples from 69 sick dogs (Groups II-IV) were selected on the basis of prior serology or PCR results. Group II consisted of 20 sick dog sample sets submitted by attending veterinarians from which no antibodies were detected using the serology panel defined below. The attending veterinarian for these cases only requested serologic testing. PCR was performed retrospectively (as described below).

Group III consisted of 25 sample sets from sick dogs that were seroreactive to at least one vector-borne pathogen. The attending veterinarian for these cases only requested serologic testing and PCR was performed retrospectively (as described below).

Group IV consisted of sample sets from 24 sick dogs that were previously PCR positive for *Babesia*, *Bartonella*, *Ehrlichia*, *Anaplasma* or *Rickettsia* species. The attending veterinarian for these cases only requested PCR testing (for one or more organisms). Both serologic testing and PCR testing, including testing for additional organisms (as described below) were performed retrospectively.

### Serology

For this study, all serum samples were tested using a commercial ELISA based kit^a^ and by IFA assays using a panel of VBDDL antigens. All antigens were grown *in vitro* or, in the case of *B. canis*, *in vivo* by personnel in the VBDDL using strains of canine or feline origin. Briefly, antibody responses to *B. canis*, *Bartonella henselae*, *Bartonella vinsonii* subspecies *berkhoffii*, *E. canis* and spotted fever group *Rickettsia* were tested by traditional IFA practices with fluorescein conjugated goat anti-dog IgG (Thermo Fisher Scientifics, Waltham MA 02452) [14].

Serum samples were diluted in phosphate buffered saline (PBS) solution containing 1% normal goat serum, 0.05% Tween-20 and 0.5% powdered nonfat dry milk (BioRad, Hercules, CA) to block non-specific antigen binding sites [15]. Seropositive samples were defined as having endpoint titers?≥?1:64 using a twofold scale of 1:16 – 1:8192. *Dirofilaria immitis* antigen, as well as antibodies to *Anaplasma* spp., *Ehrlichia* spp. and the C6 peptide of *B. burgdorferi*, were detected using a commercial in-house ELISA-based kit according to manufacturer’s instructions. Serum from the healthy dog population had been previously tested using a version of the ELISA kit^b^ which did not contain *Anaplasma* spp. antigens. Due to limited serum, results for these dogs were not determined for *Anaplasma* spp.

### PCR testing

Following selection of samples for the various groups (Groups I-IV), all EDTA-anti-coagulated blood samples were randomized and tested in an operator blinded fashion. Ten microbial genera (*Anaplasma*, *Babesia*, *Bartonella*, *Borrelia*, *Ehrlichia*, *Francisella*, hemotropic *Mycoplasma*, *Neorickettsia*, *Rickettsia*, and *Dirofilaria*) were targeted using specific PCR assays as previously reported [16-22]. To further assess potential infection with *Dilofilaria* spp. PCR amplification of *Wolbachia* spp. was used as additional indirect evidence to support a diagnosis of canine heartworm disease [21-23]. In all cases in which blood samples were PCR positive, direct DNA sequencing was performed. Reference sequences for this study included the following GenBank accession numbers: AY055469 (*Anaplasma phagocytophilum*), M82801 (*A. platys*), AY072925 (*B. canis vogeli*), AY618928 (large unnamed *Babesia* sp. “coco”) AF271081 (*B. gibsoni*), NC_005956.1 (*Bartonella henselae* Houston I), AF369529 (*Bartonella henselae* SA2), DQ059763 (*B. vinsonii* subsp. *berkhoffii* genotype II), DQ059764 (*B. vinsonii* subsp. *berkhoffii* genotype III), AF312490 (*B. koehlerae*), NC_007354 (*Ehrlichia canis*), NR_044747 (*E. ewingii*), AY529641 (*Mycoplasma haemocanis*), GQ129113 (*M. haematoparvum*) and CP000848 (*Rickettsia rickettsii*). Sequences were compared to the GenBank database using the Basic Local Alignment Search Tool.

### Statistical analysis

Agreement between PCR-based assays and serological assays was assessed by calculating an un-weighted kappa statistic using a statistical program.^c^ For agreement between PCR and serological assays for *Anaplasma*, the detection of any *Anaplasma* sp. by PCR was considered a positive result and the detection of a colorimetric change in the *Anaplasma* ELISA was considered a positive result. For agreement between PCR and serological assays for *Babesia*, the detection of any *Babesia* sp. by PCR was considered a positive result and the detection of an antibody titer?≥?1:64 against *B. canis* was considered a positive result. For agreement between PCR and serological assays for *Bartonella*, the detection of any *Bartonella* sp. by PCR was considered a positive result and the detection of an antibody titer?≥?1:64 against any *Bartonella* sp. was considered a positive result. For agreement between PCR and serological assays for *Ehrlichia*, the detection of any *Ehrlichia* sp. by PCR was considered a positive result and the detection of an antibody titer?≥?1:64 against *E. canis* or a colorimetric change in the *Ehrlichia* spp. ELISA was considered a positive result. For agreement between PCR and serological assays for *Rickettsia*, the detection of any *Rickettsia* sp. by PCR was considered a positive result and the detection of an antibody titer?≥?1:64 against *R. rickettsii* was considered a positive result. Agreement between PCR-based assays and serological assays could not be calculated for *Borrelia* spp., *Dirofilaria* sp. or *Mycoplasma* spp. Kappa values of 0–0.20 indicated poor agreement, 0.21–0.40 indicated fair agreement, 0.41–0.60 indicated moderate agreement, 0.61–0.80 indicated strong agreement, and 0.81–1 indicated almost perfect agreement.

## Results

The overall agreement between PCR and serology test results for dogs in Groups I to IV are summarized in Table 1. The number of serology versus PCR positive dogs within each Group for each pathogen is summarized in Table 2. Molecular and serological results for each pathogen and for each dog in each group are detailed in Tables 3, 4, 5 and 6. DNA of *Neorickettsia*, *Wolbachia* and *Francisella* spp. was not PCR amplified, from any dog in the study.

**Table 1:** Overall agreement between PCR and serology test results for Groups I to IV

**Group**	**Dogs**	**Positive by PCR (%)**	**Positive by Serology (%)**	**Agreement between positives (%)**
I	30	7 (23.3)	13 (43.3)	2/14 (28.6)
II	20	1 (5)	0 (0)	0/1 (0)
III	25	2 (8)	25 (100)	1/25 (4)
IV	24	24 (100)	17 (70.8)	12/24 (50)

**Table 2:** **Number of seropositive** (**Ser**) **or PCR positive dogs within each Group for each pathogen**

**Group**	**Dogs**	***Babesia***	***Bartonella***	***Rickettsia***	***Ehrlichia***	***Anaplasma***	***Borrelia***	***Dilofilaria***
	tested	Ser-PCR	Ser-PCR	Ser-PCR	Ser-PCR	Ser-PCR	Ser-PCR	Ser-PCR
I	30	0-0 [0]	6-6 [2]	7-0 [0]	1-0 [0]	N/A	0-0 [0]	0-0 [0]
II	20	0-1 [0]	0-0 [0]	0-0 [0]	0-0 [0]	0-0 [0]	0-0 [0]	0-0 [0]
III	25	0-0 [0]	6-0 [0]	12-1 [1]	8-0 [0]	3-0 [0]	4-0 [0]	0-0 [0]
IV	24	6-11 [6]	6-6 [1]	2-0 [0]	4-4 [3]	2-5 [2]	3-0 [0]	1-0 [0]

Number of dogs positive for both serology and PCR (agreement) are listed between brackets.

**Table 3:** **IPRL PCR and serology of thirty healthy dogs** (**Group I**) **from North Carolina**

		**Serology**					
**Sample #**	**IPRL PCR**	***Babesia spp****.*	***Bartonella spp****.*	***Rickettsia spp.***	***Ehrlichia spp*.**	***Borrelia burgdorferi***	***Dirofilaria immitis***
1	BhSA2	Neg	Neg	Pos	Neg	Neg	Neg
2	BhSA2	Neg	Neg	Pos	Pos	Neg	Neg
3	BhSA2	Neg	Neg	Pos	Neg	Neg	Neg
4	BhHI	Neg	Pos	Neg	Neg	Neg	Neg
5	BhHI	Neg	Pos	Neg	Neg	Neg	Neg
6	BhSA2	Neg	Neg	Neg	Neg	Neg	Neg
7	Mhc	Neg	Pos	Neg	Neg	Neg	Neg
8	Neg	Neg	Pos	Neg	Neg	Neg	Neg
9	Neg	Neg	Pos	Neg	Neg	Neg	Neg
10	Neg	QNS	Pos	Neg	Neg	Neg	Neg
11	Neg	Neg	Neg	Pos	Neg	Neg	Neg
12	Neg	Neg	Neg	Pos	Neg	Neg	Neg
13	Neg	Neg	Neg	Pos	Neg	Neg	Neg
14	Neg	Neg	Neg	Pos	Neg	Neg	Neg
15	Neg	Neg	Neg	Neg	Neg	Neg	Neg
16-30	Neg	Neg	Neg	Neg	Neg	Neg	Neg

*Abbreviations*: *IPRL* Intracellular Pathogens Research Laboratory, *BhHIBartonella henselae* Houston I, *BhSA2Bartonella henselae* San Antonio 2, *MhcMycoplasma haemocanis.*

**Table 4:** **IPRL PCR assay results in twenty sick dogs** (**Group II**) **that were IFA and Snap® 4DX kit seronegative in the VBDDL**

		**Serology**						
**Sample #**	**IPRL PCR**	***Babesia spp***	***Bartonella spp***	***Rickettsia spp.***	***Ehrlichia spp.***	***Anaplasma spp.***	***Borrelia burgdorferi***	***Dirofilaria immitis***
#1	B. gib	Neg	Neg	Neg	Neg	Neg	Neg	Neg
**#2 to #20**	Neg	Neg	Neg	Neg	Neg	Neg	Neg	Neg

*Abbreviations*: *IPRL* Intracellular Pathogens Research Laboratory, VBDDL Vector-Borne Diseases Diagnostic Laboratory, *B.gibBabesia gibsoni*.

**Table 5:** **IPRL PCR assay results for twenty five sick dogs** (**Group III**) **that were IFA or Snap® 4DX kit positive in the VBDDL**

		**Serology**						
**Sample #**	**IPRL PCR**	***Babesia spp***	***Bartonella spp***	***Rickettsia spp.***	***Ehrlichia spp.***	***Anaplasma spp.***	***Borrelia burgdorferi***	***Dirofilaria immitis***
1	R. rickettsii	Neg	Neg	Pos	Neg	Neg	Neg	Neg
2	Mhc	Neg	Neg	Neg	Neg	Pos	Pos	Neg
3	Neg	Neg	Neg	Neg	Pos	Neg	Neg	Neg
4	Neg	Neg	Neg	Neg	Pos	Neg	Neg	Neg
5	Neg	Neg	Neg	Neg	Pos	Neg	Neg	Neg
6	Neg	Neg	Neg	Neg	Pos	Neg	Neg	Neg
7	Neg	Neg	Pos	Neg	Pos	Neg	Neg	Neg
8	Neg	Neg	Pos	Neg	Pos	Neg	Neg	Neg
9	Neg	Neg	Neg	Neg	Pos	Neg	Neg	Neg
10	Neg	Neg	Neg	Pos	Pos	Neg	Neg	Neg
11	Neg	Neg	Neg	Pos	Neg	Neg	Neg	Neg
12	Neg	Neg	Neg	Pos	Neg	Neg	Neg	Neg
13	Neg	Neg	Pos	Pos	Neg	Neg	Neg	Neg
14	Neg	Neg	Pos	Pos	Neg	Neg	Neg	Neg
15	Neg	Neg	Neg	Pos	Neg	Neg	Neg	Neg
16	Neg	Neg	Neg	Pos	Neg	Neg	Neg	Neg
17	Neg	Neg	Neg	Pos	Neg	Neg	Neg	Neg
18	Neg	Neg	Pos	Pos	Neg	Neg	Neg	Neg
19	Neg	Neg	Neg	Pos	Neg	Neg	Neg	Neg
20	Neg	Neg	Neg	Pos	Neg	Neg	Neg	Neg
21	Neg	Neg	Pos	Neg	Neg	Neg	Pos	Neg
22	Neg	Neg	Neg	Neg	Neg	Neg	Pos	Neg
23	Neg	Neg	Neg	Neg	Neg	Neg	Pos	Neg
24	Neg	Neg	Neg	Neg	Neg	Pos	Neg	Neg
25	Neg	Neg	Neg	Neg	Neg	Pos	Neg	Neg

*Abbreviations*: *IPRL* Intracellular Pathogens Research Laboratory, *VBDDL* Vector-Borne Diseases Diagnostic Laboratory, *hcMycoplasma haemocanis*; *R. rickettsiiRickettsia rickettsii*.

**Table 6:** **IPRL PCR and serology assays results for twenty four dogs** (**Group IV**) **that were PCR positive based upon prior diagnostic testing in the VBDDL**

		**Serology**						
**Sample#**	**IPRL**	***Babesia spp*.**	***Bartonella spp*.**	***Rickettsia spp.***	***Ehrlichia spp*.**	***Anaplasma spp*.**	***Borrelia burgdorferi***	***Dirofilaria immitis***
1	A.ph & B.gib	Neg	Neg	Pos	Neg	Pos	Pos	Neg
2	A.ph	Neg	Neg	Neg	Neg	Pos	Pos	Neg
3	A.ph	Neg	Neg	Neg	Neg	Neg	Pos	Neg
4	A.pl	Neg	Neg	Neg	Neg	Neg	Neg	Neg
5	E.canis & B.canis	Pos	Neg	Neg	Pos	Neg	Neg	Pos
6	E.canis	Neg	Pos	Neg	Pos	Neg	Neg	Neg
7	E.ew	Neg	Neg	Neg	Pos	Neg	Neg	Neg
8	E.ew	Neg	Neg	Neg	Neg	Neg	Neg	Neg
9	B.canis	Neg	Pos	Neg	Neg	Neg	Neg	Neg
10	B.canis	Pos	Pos	Neg	Neg	Neg	Neg	Neg
11	B.canis	Neg	Neg	Neg	Neg	Neg	Neg	Neg
12	B.canis/B. coco	Neg	Neg	Pos	Neg	Neg	Neg	Neg
13	B.coco	Neg	Neg	Neg	Pos	Neg	Neg	Neg
14	B.gib	Pos	Neg	Neg	Neg	Neg	Neg	Neg
15	B.gib	Pos	Pos	Neg	Neg	Neg	Neg	Neg
16	B.gib	Pos	Neg	Neg	Neg	Neg	Neg	Neg
17	B.gib	Pos	Pos	Neg	Neg	Neg	Neg	Neg
18	BvbII	Neg	Pos	Neg	Neg	Neg	Neg	Neg
19	BvbIII	Neg	Neg	Neg	Neg	Neg	Neg	Neg
20	Bk	Neg	Neg	Neg	Neg	Neg	Neg	Neg
21	Bk & Apl	Neg	Neg	Neg	Neg	Neg	Neg	Neg
22	BhHI	Neg	Neg	Neg	Neg	Neg	Neg	Neg
23	BhHI	Neg	Neg	Neg	Neg	Neg	Neg	Neg
24	Bart sp	Neg	Pos	Pos	Pos	Neg	Neg	Neg

*Abbreviations*: *IPRL* Intracellular Pathogens Research Laboratory, *VBDDL* Vector-Borne Diseases Diagnostic Laboratory, *A.phAnaplasma phagocytophilum*, *A.plAnaplasma platys*, *E.canisEhrlichia canis*, *E.ewEhrlichia ewingii*, *B.canisBabesia canis*, *B.coco* Large unnamed *Babesia* sp. , *B.gibBabesia gibsoni*, *BvbIIBartonella vinsonii* subspecies *berkhoffii* Genotype II, *BvbIIIBartonella vinsonii* subspecies *berkhoffii* Genotype III, *BkBartonella koehlerae*, *BhHIBartonella henselae* Houston I, *BhSA2Bartonella henselae* San Antonio 2, *Bart* sp *Bartonella* species, unable to sequence.

### Group I: clinically healthy dogs

Of the 30 Group I healthy dog blood samples, 16 had no evidence of infection with or exposure to CVBDs (Table 3). Combined serological and molecular testing identified exposure to or infection with CVBDs in 14 healthy dogs. Thirteen dogs were seroreactive (antibody titer?≥?1:64) to one (n?=?12) or more (n?=?1) antigens by IFA testing. No healthy dog was IFA seroreactive to *B. canis*, or *B. vinsoni* subsp *berkhoffii* antigens. One, six, and seven dogs were seroreactive to *E. canis*, *B. henselae* or *R. rickettsii* antigens, respectively. All 30 Group I dogs were negative by ELISA, including the one *E. canis* IFA seroreactor. Based upon PCR amplification and DNA sequencing, 6 dogs were infected with *B. henselae*, of which only two were *B. henselae* seroreactive. Three *R. rickettsii* seroreactors were PCR positive for *B. henselae*, one of which was also *E. canis* seroreactive. One dog was PCR positive for *Mycoplasma haemocanis*. This dog was seroreactive against *B. henselae* antigens but did not have detectable *Bartonella* DNA in its blood.

### Group II: sick dogs in which CVBD antibodies were not detected and for which no PCR testing had been requested

Nineteen of 20 Group II sick dogs had no evidence of infection with or exposure to CVBDs (Table 4). The remaining dog was PCR positive for *B. gibsoni* and seronegative to all antigens tested. *Babesia gibsoni* IFA testing was not performed as a component of this study.

### Group III: sick dogs in which CVBD antibodies against at least one pathogen were detected and for which no PCR testing had been requested

Of the 25 dogs in Group III, two dogs were PCR positive (Table 5). One dog was actively infected with *R. rickettsii* based upon PCR and DNA sequencing and was seroreactive against *R. rickettsii* with a titer of 1:128. The other dog had antibodies against *B. burgdorferi* and *Anaplasma* spp. detected by ELISA and was PCR positive for *Mycoplasma haemocanis*. All eight *E. canis* IFA seroreactive dogs were positive by ELISA; however, none were PCR positive. Six dogs were seroreactive to *B. henselae* antigens and one to *B. vinsonii* subsp. *berkhoffii* antigens, but were not *Bartonella* PCR positive.

### Group IV: sick dogs from which DNA of at least one CVBD pathogen was detected by PCR testing, but serological testing had not been requested

Group IV, consisted of 24 dogs that were previously PCR positive with *Ehrlichia*, *Anaplasma*, *Babesia* or *Bartonella* spp. When serological and PCR assays were performed beyond the PCR requested by the attending clinician exposure to or infection with additional CVBD of different genera were identified in 13 dogs (Table 6).

### Comparative serological and molecular testing

For the PCR assays requested by the attending veterinarian there was complete agreement between VBDDL PCR results and repeat blinded testing. Overall, the agreement between PCR and serology ranged from 28.6% for the healthy dogs to 4 - 50% for sick dogs depending on groups (Table 1). The agreement between PCR and serology depended greatly on the tested species. There was strong agreement (kappa: 0.637, 95% CI 0.453-0.822) between PCR-based assays and serological assays for *Babesia* spp. There was moderate agreement (kappa: 0.353, 95% CI 0.117-0.589) between PCR-based assays and serological assays for *Anaplasma* spp. There was moderate agreement (kappa: 0.289, 95% CI 0.130-0.447) between PCR-based assays and serological assays for *Ehrlichia* spp. There was poor agreement (kappa: 0.111, 95% CI -0.081-0.304) between PCR-based assays and serological assays for *Bartonella* spp. There was poor agreement (kappa: 0.069, 95% CI -0.003-0.141) between PCR-based assays and serological assays for *R. rickettsii*.

### DNA sequencing results

For all three *Babesia* spp. (*B. canis*, *B. gibsoni* and the large unnamed *Babesia* sp.), DNA sequencing analyses documented identities between 99 and 100%, when compared to our reference sequences. Sequencing analysis identified *Anaplasma* and *Ehrlichia* spp. identities between 99.7-100% when compared to reference sequences. Infection with *R. rickettsii* was confirmed in one dog (100% identity to reference sequence). *B. henselae*, *B. koehlerae* and *B. vinsonii* subsp. *berkhoffii* DNA had 100% identity with reference sequences. Both cases of hemotropic *Mycoplasma* sp. shared 100% sequence identity with our reference sequence.

## Discussion

Development of DNA amplification techniques or other molecular approaches by which CVBD pathogens can be detected and identified to the species or strain level in diagnostic samples continues to facilitate a more rational basis for selection of treatment modalities. However, from a diagnostic perspective, it does not appear that either PCR or serological assays alone are adequate to identify infection or exposure in every case (Table 2). The results of this study demonstrate that evidence of exposure to or infection with vector-borne pathogens in dogs can be increased by using a combination of serological and PCR-based assays in parallel. Use of such comprehensive testing not only resulted in detection of exposure or infection with CVBD pathogens in a greater number of dogs, it also resulted in the identification of co-infections that would have been missed if only one testing method was used. While the antibiotic treatment history and the rationale for the submission of each sample (other than the healthy control group) was not known, each group of dogs is likely to represent a scenario in which clinical, therapeutic or public health decisions are likely to be different had the clinician submitted for both PCR and serological assays in parallel.

Group I (clinically healthy dogs) could represent dogs similar to those being screened as blood donors. Depending on individual hospital blood donor screening practices (*Rickettsia*, *Borrelia* or *Dirofilaria* exposure or infection are not typically included as exclusion criterion for canine blood donors), the use of PCR and serological assays in parallel increased the number of donors that could be excluded to 33% (10/30) compared to 23% (7/30) if either PCR or serology were used alone.

Group II (sick dogs in which CVBD antibodies were not detected and for which no PCR testing had been requested) represents a population of dogs for which only serological assays were requested by the attending veterinarian. It seems plausible to assume that after the requested testing these dogs were considered less likely to be infected with or exposed to CVBD. However, the use of PCR and serological assays in parallel would have facilitated the identification of an infection in 5% (1/20) of those cases.

Group III (sick dogs in which CVBD antibodies against at least one pathogen were detected and for which no PCR testing had been requested) represents a population of dogs for which only serological assays were requested by the attending veterinarian. The addition of PCR testing facilitated the identification of an additional organism in 4% (1/25) of these cases. It is also interesting to consider that if these clinicians had only selected PCR assays, exposure to CVBD would have been missed in 92% (23/25) of the cases.

Group IV (sick dogs from which DNA of at least one CVBD pathogen was detected by PCR testing, but serological testing had not been requested) represents a population of dogs for which only one or more PCR assay(s) were requested by the attending veterinarian. The use of more extensive PCR testing and serological assays would have identified exposure to or infection with additional CVBD (i.e. potential co-infections) in 54% (13/24) of the cases. Additionally, if the attending clinician had only requested serological assays on these samples, exposure to or infection with additional CVBD (i.e. potential co-infections) that would alter the case management would have either been missed in 58% (14/24) of cases, or CVBD would have been missed entirely in 13% (3/24) of the cases. Examples where the identification would alter the case management include a dog that had antibodies against *B. canis* but was actually PCR positive for *B. gibsoni* (i.e. Group IV #14 or #16) or a dog that had evidence of infection with *A. phagocytophilum* and *B. burgdorferi* and exposure to or infection with *R. rickettsii* but was also PCR positive for *B. gibsoni*. In some cases the additional information would not have changed case management. An example of such is the dog that was IFA seroreactive against *E. canis* yet PCR positive for *E. ewingii*. Treatment for ehrlichiois may not have been started based upon seroreactivity, but would have been indicated on the basis of the PCR result.

There are several specific reasons for lack of agreement between PCR-based and serological assays, but differences in sensitivity and specificity between assays are of primary importance for interpretation. Specific examples that will result in differences include timing of sample collection, biological behavior of the pathogen and variation in host immune responses to antigens. As our understanding of CVBD and individual host responses to infection increases it is becoming clearer that our currently available diagnostic assays all have shortcomings.

## Conclusions

Cumulatively, the results of this study suggest that the use of PCR and serological assays in parallel is likely to increase the detection of infection with or exposure to CVBDs. This clinical scenario is not unknown to the veterinary profession. For example, veterinarians are comfortable accepting that a multi-modal approach, including antigen and antibody testing combined with diagnostic imaging, is ideal to diagnose feline heartworm infection [26]. However, it is important to note that the increased detection of infection with or exposure to CVBD does not always confirm disease causation and the attending veterinarian is still responsible for interpretation of test results in light of the patient’s clinical signs, laboratory abnormalities and response to treatment. Clinicians must consider the prevalence of each sign or laboratory abnormality with each type of infection and a failure to respond to appropriate treatment should prompt the consideration of alternative diagnoses. In conclusion, it still appears that an ultimate test for the diagnosis of CVBDs remains elusive. To optimize clinical decision making, clinicians should consider using panels that include serological and PCR assays in parallel to maximize their chances of detecting infection or exposure to CVBD pathogens.

### Endnotes

^a^Snap® 4DX kit, IDEXX Laboratories Inc., Westbrook, Maine; ^b^Snap® 3DX kit, IDEXX Laboratories Inc., Westbrook, Maine; ^c^WinEpiscope 2.0, http://www.clive.ed.ac.uk/.

## Abbreviations

CVBD: Canine vector-borne diseases; IFA: Indirect fluorescent antibody; ELISA: Enzyme-linked immunoabsorbent assays; VBDDL: Vector-Borne Disease Diagnostic Laboratory; IPRL: Intracellular Pathogens Research Laboratory.

## Competing interests

Drs. Birkenheuer, Breitschwerdt, and Maggi are co-directors of the VBDDL. Dr. Maggi oversees molecular testing in the VBDDL and IPRL. Ms. Hegarty supervises serological testing in the VBDDL and cultivates all antigens used in the IFA assays reported in this study. Ms. Bradley performed the serological testing. All authors contributed to the intellectual content of this manuscript.

## Authors’ contributions

RGM, AJB and MGL performed the PCR testing of the patient samples, performed DNA sequencing and alignments. JMB and BCH assisted in sample acquisition and serological testing. EB and AJB coordinated various aspects of the investigation and helped to draft the final manuscript. All authors read and approved the manuscript.
